# Genetic Variability and Population Structure of Polish Konik Horse Maternal Lines Based on Microsatellite Markers

**DOI:** 10.3390/genes12040546

**Published:** 2021-04-09

**Authors:** Agnieszka Fornal, Katarzyna Kowalska, Tomasz Zabek, Agata Piestrzynska-Kajtoch, Adrianna D. Musiał, Katarzyna Ropka-Molik

**Affiliations:** Department of Animal Molecular Biology, National Research Institute of Animal Production, 32-083 Krakow, Poland; katarzyna.kowalska@iz.edu.pl (K.K.); tomasz.zabek@iz.edu.pl (T.Z.); agata.kajtoch@iz.edu.pl (A.P.-K.); adrianna.musial@iz.edu.pl (A.D.M.); katarzyna.ropka@iz.edu.pl (K.R.-M.)

**Keywords:** Polish Konik, STR, founding mares, biodiversity, population genetics

## Abstract

**Simple Summary:**

The aim of this study is to reveal the genetic variability and population structure of maternal lines of the Polish Konik—a native Polish horse breed of the primitive type included in the Polish conservation programme. The analysis was carried out on the basis of 17 microsatellite markers routinely used for parentage testing. The structural analysis indicated the likelihood of three genetic clusters (using the Structure and Structure Harvester software). F-statistics indicated a low level of inbreeding. All mean population coefficients were close to those obtained for representatives of male founder lines. The population seemed to be stable. However, due to a previous bottleneck event, breeding strategies should focus on avoiding inbreeding depression, preventing the decrease of genetic variability, and sustaining the active female lines.

**Abstract:**

The aim of the conservation programme is to maintain the population size of endangered livestock breeds of less economic importance at a level that ensures the survival of the breed, the preservation of genetic diversity, and the preservation of as many pedigree lines as possible. The Polish Konik, a native Polish primitive-type horse breed and is one of the breeds included in such a programme in Poland. Presently, there are only 16 (of the 35 maternal lines known in 1962), some of which are endangered. We examined the genetic variability and structure of the Polish Konik maternal lines (176 individuals) on the basis of the pedigree data and 17 microsatellite markers (STRs) from parentage testing. The overall mean number of alleles was 7.647 (±0.411), the effective number of alleles was 3.935 (±0.271), the mean number of alleles for which the frequency was equal to or lower than 5% was 4.471 (±0.286), and the mean information index was 1.506 (±0.087). The structure of the population and admixture patterns were calculated with the Structure and Structure Harvester software. The structural analysis indicated three likely genetic clusters; as the most optimal K value was estimated as 3, with ∆K of 15.4188. The F-statistics results indicated a low level of inbreeding (average inbreeding coefficient F_IT_ was 0.0188, coefficient of differentiation F_ST_ was 0.0304, and mean inbreeding index value F_IS_ was −0.0119). Variability monitoring should be carried out in order to avoid inbreeding depression, while breeding strategies should be designed to prevent the decrease of genetic variability in the Polish horse breed and to sustain the active female lines.

## 1. Introduction

**Figure 1:**
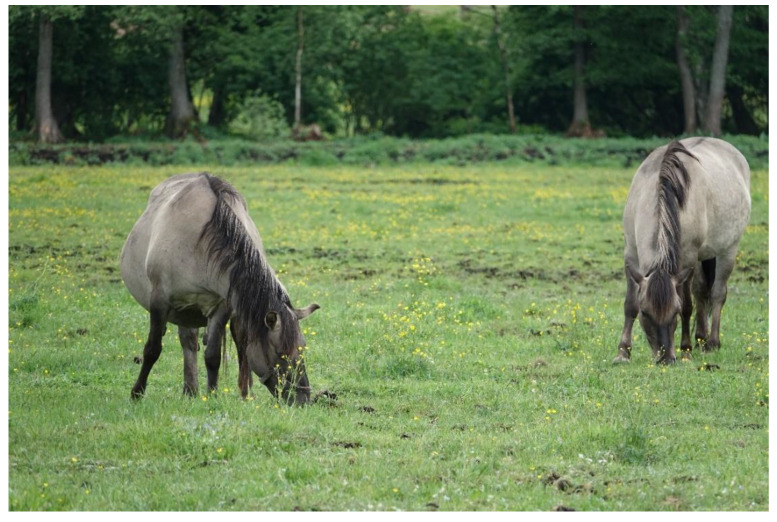
Polish Konik (photo by Izabela Dojlida).

The Polish Konik horse is one of the horse breeds covered by the conservation programme in Poland, which considers six horse breeds: Polish Konik, Hucul horse, Wielkopolska, Malopolska, Silesian, and Polish Coldblood (sokolski and sztumski types). The Hucul horse and Polish Konik studbooks were closed in the 1980s; therefore, these breeds have been kept pure, and the admixture of other breeds has been forbidden [1,2]. The Polish Konik horse (*Equus caballus*)—sometimes called Polish primitive [3] or Polish pony [4]—is a native Polish breed. The history of the breed extends nearly 100 years. This horse breed is thought to be most closely related to the extinct Tarpan, among other breeds [5]. The last representatives of the Tarpans were domesticated in the early 19th century and, after that, crossbred with domestic horses [6]. Polish Konik horses (Figure 1) have many Tarpan traits such as clear social hierarchy, high fertility, disease resistance, excellent feed efficiency, and ease in adaptation to extreme environmental conditions [7]. Polish Konik mares from the reserve Popielno including Lalka (21 progeny; lifespan, 33 years), Niwa (20 progeny; lifespan, 29 years), and Ożyna (24 progeny; age, 25 years) should be mentioned as examples of long-lived and highly fertile horses [2,8]. Polish Konik horses are generally bred under two management conditions: traditional stable housing and maintenance in a natural reservation in semi-feral/free-roaming groups [2,9]. They may be kept in reserves with the aim of maintaining their natural form of behaviour and welfare.

World War II had a ruinous impact on the breeding of Polish horses [10] including the Polish Konik breed. Nevertheless, the breed has been successfully rebuilt [5,11,12]. In 2019, according to the Polish Horse Breeders Association, the number of breeding Polish Koniks was over 1700 horses, including 1540 mares and 166 stallions [13].

Presently, Polish Konik are broadly used for hippotherapy, agritourism, and recreational riding [2], as well as for landscape shaping and biodiversity restoration in post-agricultural or forest areas. The Polish Konik breed was reintroduced to landscape maintenance in Great Britain, France, Belgium, and Germany [11], and their breeding was also extended to Sweden, Ireland, Lithuania, and the Czech Republic [14]. Year-round Konik horse grazing is beneficial for plants, as well as for rare bird populations [11]. The breed’s characteristic feature is grullo pigmentation having a mouse-grey coat with a black stripe along their back [15].

The first studbook of the breed, which was published in 1962, included 35 maternal and 6 paternal lines. In the 1980s, one more female line—Geneza—was implemented into the conservation programme. Unfortunately, 19 of maternal lines became extinct [16]. Moreover, the breeding activity of the residual lines was not sustainable. In the current population (individuals in 2019), the most numerous are the lines represented by Tarpanka I (18.4%), Traszka (15.5%), Zaza (13.1%), Liliputka I (12.7%), Karolka (12.2%), and Urszulka (7.8%), which total to 79.7%. The contributions of the 10 remaining female lines are smaller, reaching up to 5% each. Some of them are endangered: Białka (1.3%), Misia II (1.2%), Ponętna (0.8%), Geneza (0.5%), and Bona (0.4%). Furthermore, in comparison to 1995, some of the dam lines demonstrated stagnation: Liliputka I, Tygryska, Karolka, Urszulka, Popielnica, Tarpanka, and Misia II. However, some of the maternal lines were characterised by clear progression: Wola, Białka, Dzina I, Tunguska, Traszka, and Bona. Unfortunately, the lines Zaza and Ponętna showed slight regression. On the contrary, all male lines (Chochlik, Glejt I, Goraj, Liliput, Myszak, and Wicek) demonstrated sufficient breeding activity. In 2019, the most numerous male lines were Wicek (34.4%), Chochlik (18.2%), and Goraj (17.2%). Importantly, the Liliput line—which, in 1995, was considered an expired population—has been rebuilt and now represents 11.0% [17].

The breed was included in the Global Strategy for the Management of Farm Animal Genetic Resources (Food and Agricultural Organisation). Genetic diversity monitoring is crucial for livestock management, especially for small, endangered livestock breeds. A recent study of the genetic diversity of the Polish Konik showed that the inbreeding coefficient increased from 4.8% in 1980 to 8.6% in 2011 [1]. Covering the period 1980–2011, the Polish Konik population had a stable trend for founder genome equivalent (around 5) and effective population size (from 23–37). Moreover, Cieslak et al. characterised the high genetic diversity in Polish Konik maternal lines using mitochondrial DNA polymorphism. They detected 19 mtDNA haplotypes, containing 5 putatively novel and 14 identical to those previously deposited in the GenBank database, derived from other horse breeds. The newly identified haplotypes differ from others with rare single-nucleotide polymorphisms. For five maternal lines (Bona, Dzina I, Geneza, Popielica, and Zaza), they observed only one mtDNA haplotype, whereas, among the 11 remaining dam lines, segregation of 2–5 haplotypes was observed. Furthermore, single mares from Liliputka and Karolka lines had been shown to carry 4 unique haplotypes [18].

Polish Konik horses are unique. They must be preserved for future breeding works as a specific reservoir of genetic resources [12]. This study aimed to examine the genetic variability and structure of the Polish Konik breed among maternal lines and was the second part of our research on Polish Konik.

## 2. Materials and Methods

We examined the genetic variability, and population structure of the Polish Konik breed within all 16 active maternal lines.

We verified maternal lines based on pedigree data and identified all 16 maternal lines. We randomly selected representative samples of maternal lines from our database. Samples with doubtful or excluded pedigree were eliminated. As a result of a random selection among the selected samples, representatives for fifteen of the sixteen lines were found; therefore, the representatives of the missing line were selected manually. Finally, we tested 176 horse samples representing the sixteen maternal lines, where the distribution of the individuals among the lines was as follows: Białka (4 individuals), Dzina I (14), Geneza (3), Karolka (15), Liliputka I (15), Misia II (3), Ponętna (8), Popielica (15), Tarpanka I (14), Traszka (15), Tunguska (15), Tygryska (12), Urszulka (15), Wola (15), Zaza (14), and Bona (2). We used peripheral blood and hair follicle samples collected for routine horse parentage testing (a collection of DNA samples from different regions of Poland) and the panel of 17 microsatellite markers, which was currently recommended by the International Society for Animal Genetics (ISAG).

Isolation, using a Wizard Genomic DNA Purification Kit (Promega, WI, USA) or Sherlock AX (A&A Biotechnology, Gdynia, Poland) and from hair follicles using Sherlock AX (A&A Biotechnology), were conducted as in our previous study. Amplification of multiplex reactions, electrophoresis conditions, and fragment analysis were carried out with the same methods as described in our previous report [19]. The 17 markers recommended by ISAG were regularly checked, validated, and standardised by ISAG horse parentage comparison tests [19].

We used the Structure 2.3.4 software [20] to analyse the genetic structure of the Polish Konik maternal lines. We set the same parameters for the clustering methods as in our previous report regarding Polish Konik paternal lines [19]. In the procedure, we set the number of clusters (K) from 1 to 20 and then estimated ∆K using the Structure Harvester v0.6.94 software [21].

We estimated the genetic diversity using GenAlEx6.5 [22,23], FSTAT v2.9.3 [24], and the pegas and adegenet packages for R [25,26]. We also used the software described by Radko et al. [27] to estimate the power of discrimination (PD), the combined probability of exclusion (PE), the probability of exclusion for each locus for one of the known parental genotypes (PE_1_) and both parental genotypes (PE_2_), and the probability of identity (P_ID_).

## 3. Results

The results showed that the seventeen microsatellite loci analyses for 176 Polish Konik samples represented all sixteen maternal lines. We identified the overall mean number of alleles as 7.647 (±0.411), where the effective number of alleles was 3.935 (±0.271) for all samples. The mean number of alleles with a frequency equal to or lower than 5% was 4.471 (±0.286). The mean information index was 1.506 (±0.087).

**Figure 2:**
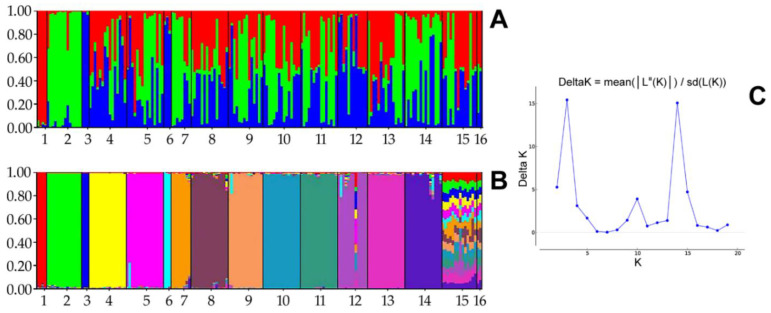
Structural analysis for K = 3 (**A**) and K = 14 (**B**) of sixteen Polish Konik maternal lines based on the ∆K method: 1, Białka; 2, Dzina I; 3, Geneza; 4, Karolka; 5, Liliputka I; 6, Misia II; 7, Ponętna; 8, Popielica; 9, Tarpanka I; 10, Traszka; 11, Tunguska; 12, Tygryska; 13, Urszulka; 14, Wola; 15, Zaza; and 16, Bona. (**C**) ∆K estimated from 1 to 20 in ten independent simulations for every K. The highest likelihood and ∆K were observed for K = 3 and K = 14.

We tested the population structure and admixture pattern within maternal lines using the Structure 2.3.4 software. We used the same running length as in our previous research and set the number of clusters (K) from 1 to 20, with ten independent simulations for every K. Next, we identified the most likely K through ∆K using the Structure Harvester v0.6.94 software to investigate the genetic groups in the data set. The structural analysis results are presented in Figure 2. The optimal K value was estimated to be 3, where ∆K was estimated as 15.4188, a slightly lower value of ∆K (15.0639) was obtained with K equal to 14.

**Figure 3:**
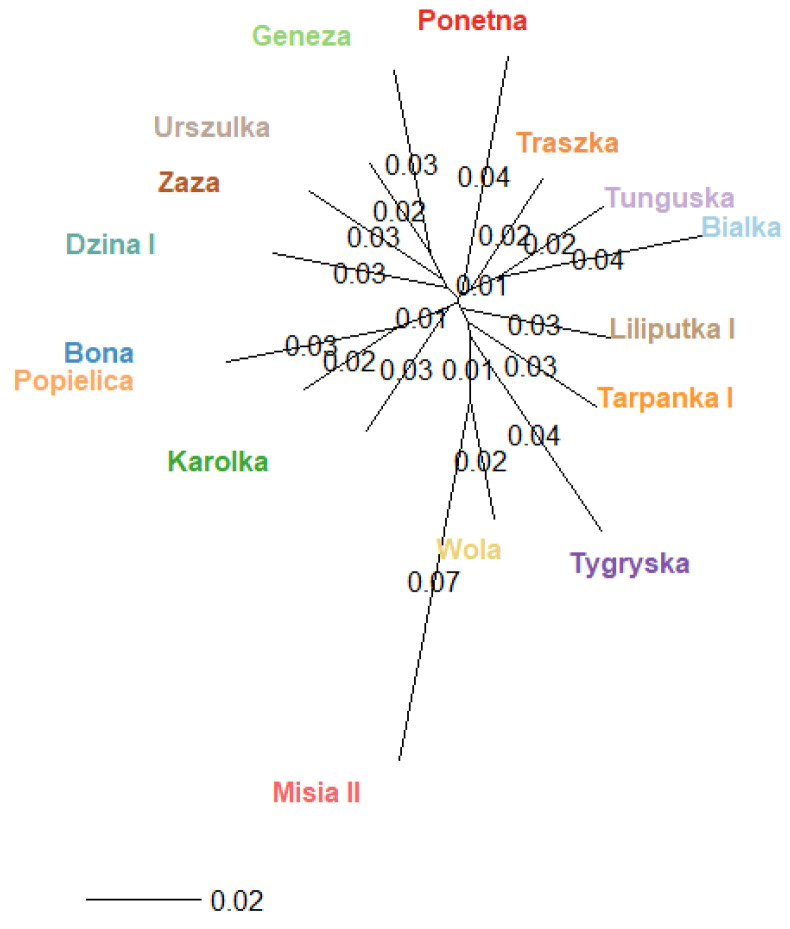
Population structure of 16 dam lines–unrooted Nei tree based in the Fst distant matrix.

In the structural analysis, three and fourteen inferred clusters best suited the data set, considering the highest ∆K values. The results were not unambiguous and did not answer the question of how many genetic clusters there were. To additionally visualise the relation among dam lines, we calculated the Fst values between pairs of populations based on Nei’s estimator using the adegenet package for R [26], which are presented in Figure 3.

The average allelic richness estimated for each locus for all samples was 7.635. Allelic richness per locus (<5.000) was the lowest at loci HTG6 (4.956) and HTG7 (4.978), and the highest (>8.000) were at ASB2, HTG10, VHL20, ASB17, and ASB23 (from 8.983 to 10.989). The allele’s mean number for the whole group studied was 7.647 (±0.411), while the effective allele’s mean number was 4.471 (±0.286).

Genetic structure coefficients, as described by F-statistics, are presented in Table 1. The population differentiation fixation index (F_ST_) was low, the mean inbreeding coefficient (F_IT_) was close to zero (0.0188), while the mean value of inbreeding index was negative (−0.0119). The coefficients describing the population were then calculated (Table 2).

The mean value of expected heterozygosity (0.7111) was higher than the observed heterozygosity (0.7019). The power of discrimination and exclusion were nearly 1 (99.9999% and 99.9989%, respectively). The polymorphism information content (PIC) was close to or higher than 0.7 for a majority of microsatellite (STR) markers in the studied horse population (except for HTG4, HTG7, and HTG6). The cumulative values of the remaining coefficients were high for both probability of exclusion and power of discrimination as well the cumulative parentage exclusion probability, when one (CPE_1_) or both (CPE_2_) parents were known. The precise values of those coefficients were 99.9321% and 99.9996%, respectively.

## 4. Discussion

Monitoring the genetic structure and diversity of a small population is essential for maintaining its diversity and the uniqueness of the gene pool. This is imperative in terms of the unique natural-breeding relict, which is the Polish Konik. The present report is a continuation of a previous study concerning the genetic variety in founding sire lines [19]. In the present study describing the Polish Konik genetic structure, we focused on the maternal lines, thus providing a complete view of the population structure. The number of alleles was a little higher than that obtained in our previous study (6.157 in the data set representing paternal lines [19] versus 7.647 for samples representing maternal lines in this study). The sum of alleles for whole loci in the examined data set was higher (146 alleles in the whole set) than that in the data set used in previous research (122). The lowest number of alleles was detected in HTG6 (five variants versus three allelic variants presented in this loci in the previous study).

Our structural analysis results of the maternal line were ambiguous. Our research aimed to assign individuals to clusters identified by using the Bayesian algorithm to estimate if they corresponded to the number of maternal lines indicated in the literature [17]. Ten independent simulations for each of K = 2 to K = 20 were used to identify the most likely K describing our data set. The optimal value was estimated as K = 3. Thus, the structural analysis indicated that there may be three genetic clusters, corresponding to the genetic cluster number obtained for the paternal lines (highest for two and three ∆K [19]). A slightly lower ∆K was obtained for fourteen genetic clusters, which may correspond to the putative number of maternal lines for our data set. A certain bias in our results could be due to probable pedigree inconsistency for a single individual from line number 12 (i.e., the Tygryska line). After a deep pedigree verification, we supposed that this individual presumably did not belong to this maternal line. Probable error in the pedigree may occur at the stage of recording the putative parents in the pedigree when the control of the pedigrees was not carried out. Fourteen genetic clusters, as a less likely variant in our study, did not correspond to the number of maternal lines, which could be interpreted as the number of dam line founders potentially being lower. Other explanations for similarities in maternal lines could be an eventual common origin (i.e., if some founders were related). However, K = 14 was less likely than K = 3, such that this alternative scenario was only speculative. The low number of genetic clusters generated using our sample collection representing active dam lines may not be descriptive for the whole population of Polish Konik derived from those lines. The more probable number of genetic clusters in our study was three, consistent with the Bayesian clustering of paternal lines [19]; although the outcomes of the structural analyses conducted using the Structure software depended on the marker used, the number of loci, the number of individuals per population (group), and the number of individuals in populations. The genetic structure may not be representative and cannot always correspond to the whole breed population [28]. Nevertheless, we prepared our data set representing the maternal lines with the greatest possible care.

The F-statistic coefficients were similar to those estimated in our previous study for the paternal line representatives: F_ST_ (0.0304) and F_IT_ (0.0188) were close to zero. The mean F_IS_ was also negative (−0.0119), indicating a low level of inbreeding. The mean H_o_ (0.7019) in this study was a little higher than that calculated for paternal representatives (0.6519), but the general value of heterozygosity in both research works was the same. H_o_ for X-linked LEX3 was higher in this study for obvious reasons. The PIC values were generally high (except for HTG4, HTG7, and HTG6). All remaining mean population coefficients were overall the same as for representatives of male founder lines [19].

We also obtained similar coefficients to the results presented by Gralak et al. [29] and Mackowski et al. [1] for 12 STRs, as in previous research. In comparison to the other research, based on 17 STRs for sixteen Polish Konik dam lines (with a total number of 94 individuals representing all lines—16 female lines remained), we obtained slightly higher mean values, certainly resulting from the size of the data set presented by Szwaczkowski et al. [30]. Slightly higher population coefficients were obtained, based on the same microsatellite markers set reported by Szwaczkowski et al., the H_o_ and H_e_ (0.6225 and 0.6837, respectively), as well as the PIC value (0.6512) [30]. We also obtained quite similar results to the same coefficients reported by Castaneda et al. [31] for Polish Koniks. The origin of the samples (country) used by these authors was not known, and they did not use the same set of microsatellites. Thus, a comparison of similarities between the overall mean number of alleles, the effective number of alleles, observed and expected heterozygosities, and mean inbreeding index value would be for illustrative purposes only.

Another Polish breed covered by the conservation programme is the Hucul horse. Polish Konik and Hucul horses are sometimes compared. Both breeds are regional and native with historical similarities; both breeds are at risk of extinction (SDG local risk status) and, as endangered horse breeds, have been maintained for many years [32,33]. Furthermore, the numbers of their populations are similar (in 2019, according to the Polish Horse Breeders Association, the number of individuals was about 1600 horses, including 1446 mares and 155 stallions) [13]. The CPE_1_ and CPE_2_ coefficients of the Hucul horse are also high [34], similar to the results obtained for Polish Konik in this study. The H_o_ and H_e_ reported by Mackowski et al. [1] were also the same. Similarities of genetic diversity values for both breeds were presented by Castaneda et al. [31]. The same levels of H_o_ and H_e_ were reported for Romanian Hucul horses [32] or Hucul horses from Hungary [35].

Our study, together with our previous report [19], allowed for a comprehensive evaluation of the Polish Konik population including both male and female founding lines, based on STR markers. The obtained data is expected to be informative and helpful in the assessment of genetic diversity for the Polish Konik breed.

## 5. Conclusions and Future Approaches

Our study was carried out on the STR set currently recommended by the ISAG, where fourteen of them have been recommended by the Food and Agriculture Organization (FAO) for molecular generic characterisation. We analysed all maternal line representatives in order to illustrate that their genetic diversity is still not numerous as an endangered breed. The Polish Konik population experienced a bottleneck event, in which the number of dam lines was reduced from 35 to 16. Genetic variability and polymorphism, assessed on the basis of maternal line representatives, confirmed no significant inbreeding in the analysed Polish Konik population. However, monitoring of variability should be carried out in order to avoid inbreeding depression. Breeding strategies should be designed to prevent a lack of sustainable continuation, to continually develop active female lines, and to prevent the decrease of the genetic variability in the Polish Konik horse breed. Using molecular markers to assess genetic diversity can be a reasonable tool for use in conservation programmes. The results obtained should be supplemented in the future through research concerning mtDNA, as well as variability on the Y chromosome, to obtain a more complete view of the founding lines.

## Figures and Tables

**Table 1:** Genetic structure coefficients for each locus (F-statistics).

	F_IT_	F_ST_	F_IS_
**AHT4**	−0.0238	0.0359	−0.0619
**AHT5**	−0.0358	0.0199	−0.0568
**ASB2**	0.0039	0.0272	−0.0240
**HMS2**	−0.0003	0.0316	−0.0329
**HMS3**	−0.0160	0.0350	−0.0529
**HMS6**	0.0394	0.0141	0.0257
**HMS7**	−0.0260	0.0103	−0.0367
**HTG10**	0.0158	0.0241	−0.0085
**HTG4**	−0.0136	0.0385	−0.0542
**HTG6**	0.0416	0.0388	0.0029
**HTG7**	0.0649	0.0340	0.0320
**VHL20**	−0.0258	0.0265	−0.0538
**ASB17**	−0.0046	0.0415	−0.0481
**ASB23**	0.1524	0.0446	0.1129
**CA425**	0.0379	0.0172	0.0211
**HMS1**	0.0573	0.0516	0.0060
**LEX3**	0.0518	0.0259	0.0265
**Mean**	0.0188	0.0304	−0.0119

F_IT_—inbreeding coefficient of an individual relative into the whole population; F_ST_—coefficient of differentiation, fixation index; F_IS_—deviation from Hardy–Weinberg proportions (within-population inbreeding coefficient).

**Table 2:** Population coefficients of the 17 microsatellite markers (STR set).

	H_o_	H_e_	PD	PE	PIC	PE_1_	PE_2_	P_ID_
**AHT4**	0.7821	0.7600	0.8945	0.5663	0.7240	0.3671	0.5459	0.0936
**AHT5**	0.8156	0.7843	0.9179	0.6284	0.7513	0.4010	0.5791	0.0795
**ASB2**	0.7933	0.7976	0.9322	0.5867	0.7766	0.4467	0.6256	0.0620
**HMS2**	0.7486	0.7446	0.8961	0.5074	0.7096	0.3484	0.5298	0.1002
**HMS3**	0.7989	0.7822	0.9088	0.5970	0.7506	0.4010	0.5801	0.0790
**HMS6**	0.7598	0.7878	0.9219	0.5266	0.7550	0.4038	0.5822	0.0779
**HMS7**	0.7151	0.6945	0.8508	0.4520	0.6410	0.2786	0.4452	0.1468
**HTG10**	0.8101	0.8193	0.9407	0.6178	0.7956	0.4717	0.6450	0.0564
**HTG4**	0.5307	0.5208	0.7449	0.2158	0.4902	0.1507	0.3172	0.2602
**HTG6**	0.1788	0.1855	0.3175	0.0243	0.1765	0.0173	0.0940	0.6724
**HTG7**	0.5978	0.6359	0.8081	0.2882	0.5740	0.2158	0.3713	0.1944
**VHL20**	0.8101	0.7861	0.9205	0.6178	0.7564	0.4149	0.5920	0.0755
**ASB17**	0.8045	0.7962	0.9159	0.6074	0.7700	0.4366	0.6129	0.0677
**ASB23**	0.6145	0.7205	0.8788	0.3087	0.6741	0.3082	0.4815	0.1245
**CA425**	0.7095	0.7344	0.8959	0.4431	0.7042	0.3468	0.5307	0.1007
**HMS1**	0.6983	0.7359	0.8879	0.4257	0.6940	0.3337	0.5094	0.1116
**LEX3**	0.7654	0.8033	0.9300	0.5364	0.7758	0.4384	0.6150	0.0662
	0.7019 *	0.7111 *	0.9999 **	0.9999 **	0.6776 *	0.9993 **	0.9999 **	3.79 × 10^−17^ **

H_o_, H_e_—observed and expected heterozygosities, respectively; PD—power of discrimination; PE—combined probability of exclusion; PIC—polymorphic information content; PE_1_, PE_2_—the probability of exclusion for each locus for one of the known parental genotypes and for both parental genotypes, respectively; P_ID_—probability of identity; *—mean value; **—cumulative value.

## Data Availability

Not applicable.
